# Trombólise na Embolia Pulmonar: Octogenários Merecem mais Atenção!

**DOI:** 10.36660/abc.20210912

**Published:** 2022-01-01

**Affiliations:** 1 Universidade Estadual Paulista Júlio de Mesquita Filho Faculdade de Medicina Campus de Botucatu - Clínica Médica Botucatu SP Brasil Universidade Estadual Paulista Júlio de Mesquita Filho Faculdade de Medicina Campus de Botucatu - Clínica Médica, Botucatu, SP - Brasil

**Keywords:** Insuficiência Cardíaca/mortalidade, Embolia Pulmonar, Anticoagulantes/uso terapêutico, Terapia Trombolítica, Hemorragia, Idoso de 80 Anos ou Mais

A embolia pulmonar (EP) é uma doença cardiovascular comum que pode ser potencialmente fatal, e sua incidência aumenta com a idade. Ela é a terceira causa mais comum de mortalidade cardiovascular, além de ser responsável por 100.000 a 180.000 mortes por ano.^1^ A insuficiência cardíaca do lado direito e recorrências são as principais causas de morte associadas à EP. A maioria das mortes ocorre na primeira hora após os pacientes apresentarem instabilidade hemodinâmica. Portanto, o prognóstico desses pacientes depende de um tratamento rápido.^1,2^

Para determinar uma abordagem terapêutica adequada em pacientes com EP, a estratificação de risco apropriada é decisiva para iniciar o tratamento eficaz o mais rápido possível e evitar mortes.^2^ Portanto, pacientes com baixo risco de mortalidade podem ser candidatos ao tratamento com anticoagulantes em casa ou à alta hospitalar antecipada. Pacientes com risco intermediário podem demandar terapias antitrombóticas e observação clínica próxima para escalar o regime terapêutico no caso de debilitação clínica. Já os pacientes com alto risco devem realizar a pronta revascularização direcionada por cateter percutâneo ou farmacológico ou por tratamento cirúrgico.^2^

A trombólise combinada com anticoagulação padrão tem potencial de salvar vidas. Esses procedimentos levam a melhorias mais rápidas na perfusão pulmonar, desequilíbrio pulmonar, troca gasosa, e disfunção do ventrículo direito. O principal benefício é observado quando a trombólise é administrada em 48 horas após o aparecimento dos sintomas. Sua eficácia diminui significativamente após 7 dias, mas pode trazer benefícios até 14 dias após o aparecimento dos sintomas.^3^

A vantagem clínica da terapia trombolítica foi demonstrada em pacientes com EP massiva. Pacientes de alto risco que receberam terapia trombolítica tinham um risco mais baixo de mortalidade global e relacionada a EP do que pacientes de alto risco que não a receberam.^2,3^

Entretanto, a meta-análise realizada por Quezada et al.^4^ demonstrou que, apesar da instabilidade dinâmica, apenas 23% dos pacientes de alto risco foram submetidos a trombólise.

Na terapia trombolítica, o dilema mais significativo é o risco de hemorragia. Em pacientes idosos, as comorbidades e o uso de múltiplos medicamentos aumenta ainda mais o risco de hemorragia.^4^ A situação restringe a indicação total de trombolíticos pelos clínicos, e, consequentemente, há poucos estudos robustos que avaliaram a eficácia e a segurança da trombólise nos pacientes muito idosos (>80 anos) com EP.

Nessa questão, Zengin et al.^5^ em um estudo coorte retrospectivo, avaliaram a eficiência e a segurança do tratamento por trombólise em 148 pacientes octogenários com EP. Uma dose convencional de ativador do plasminogênio tecidual (tPA) foi o agente lítico padrão usado para pacientes que compareceram em até 14 dias do aparecimento dos sintomas.^2^ Nesse estudo, os autores demonstraram um índice de mortalidade no hospital considerável de 19%. O índice para o grupo trombolítico era significativamente mais baixo em comparação ao grupo não trombolítico (10,5% *vs* 24,2%, p=0,03). Por outro lado, as complicações de hemorragia geral eram significativamente mais frequentes no grupo trombolítico, determinadas principalmente por pequenas hemorragias (35% *vs* 13%, p<0,01). Não houve diferenças ao se considerar os grandes eventos de hemorragia (7% *vs* 5,5%, p=0,71).

De forma semelhante, um estudo coorte retrospectivo,^6^ que incluiu pacientes com EP acima de 65 anos de idade, relatou a terapia trombolítica associada a mortalidade mais baixa e índices aceitáveis de complicações por hemorragia. Esses achados podem incentivar os clínicos a administrar terapia trombolítica com cuidado em pacientes idosos com EP.

Entretanto, é necessário considerar que a EP é uma doença potencialmente fatal que se torna ainda mais séria na população idosa com índices consideráveis de mortalidade no hospital. Uma parte dessa mortalidade poderia ser reduzida pela indicação cuidadosa da trombólise. É possível que a indicação de trombólise para a população idosa possa não ser restringida apenas em instabilidade hemodinâmica, mas essa questão deve ser analisada. A identificação de pacientes idosos que estão hemodinamicamente estáveis no diagnóstico, mas têm risco alto de complicações precoces, é um desafio maior.

É importante considerar que em ambos os tipos de estudos, os de estratificação de risco em pacientes com EP usando o índice de gravidade da embolia pulmonar (PESI) e sua forma simplificada (sPESI) e os estudos com sPESI mais Troponina como marcador baseiam-se principalmente em dados de uma população geral, em que os pacientes muito mais velhos eram sub-representados.^7–10^ Portanto, a aplicabilidade de uma ferramenta de estratificação de risco para pacientes idosos, desenvolvida com base em dados de populações mais jovens, é questionável. Ferramentas melhores de estratificação de risco devem ser consideradas para melhorar o tratamento clínico de pacientes idosos (>65) e muito idosos (>80) com suspeita de EP.

Outro achado interessante a se destacar nesse estudo^11^ é uma amostra que inclui apenas 30% de pacientes do sexo masculino. Embora não existam estudos específicos de comparação entre sexos que tenham avaliado a eficácia e a segurança da trombólise, é importante enfatizar que há diferenças na biologia das plaquetas e nas reações de coagulação que são associadas ao sexo, levando a resultados diferentes incluindo complicações por hemorragia.^11^ Portanto, o entendimento das diferenças relacionadas a sexo em relação à terapia antitrombótica e à anticoagulação deve levar a uma abordagem terapêutica individualizada para a prevenção e o tratamento de doenças cardiovasculares diferentes.

Contraindicações tradicionais à terapia trombolítica devem ser consideradas relativas no cenário de EP de alto risco, com o equilíbrio entre risco e benefício sendo ponderado caso a caso, especialmente nos pacientes acima dos 80 anos de idade. A indicação de uma trombólise individualizada, notadamente na população muito idosa e frágil, deve ser analisada para melhorar eficácia e segurança, levando o sexo do paciente em consideração.

Para reduzir o risco de hemorragia em octogenários, opções de trombólise com dose reduzida de agentes trombolíticos e doses mais baixas por trombólise direcionada por cateter nessa população precisam ser mais estudadas.
