# Supplementary material for: Trombólise na Embolia Pulmonar: Octogenários Merecem mais Atenção!
Source: Arq Bras Cardiol. 2022 Jan 1;118(1):75–6. [Article in Portuguese] doi: 10.36660/abc.20210912 (PMC8959059; doi:10.36660/abc.20210912)
Supplement: Translation

# Translation: Thrombolysis in Pulmonary Embolism: Octagenarians Deserve More Attention!

**Affiliations:** 1 Botucatu SP Brazil Universidade Estadual Paulista Júlio de Mesquita Filho Faculdade de Medicina Campus de Botucatu - Clínica Médica, Botucatu, SP - Brazil

**Keywords:** Heart Failure/mortality, Pulmonary Embolism, Anticoagulants/therapeutic use, Thrombolytic Therapy, Hemorrhage, Aged 80 and Over

Pulmonary embolism (PE) is a common cardiovascular disease that may be life threatening, and its incidence increases with age. It is the third most common cause of cardiovascular mortality and is also responsible for 100,000 to 180,000 deaths per year.^1^ Right-sided heart failure and recurrences are the main causes of death associated with PE. Most deaths occur within the first hour of patients presenting hemodynamic instability. Therefore, the prognostic of those patients depends on rapid treatment.^1,2^

To determine a suitable therapeutic management approach in patients with PE, the appropriate risk stratification is decisive in order to begin effective treatment as early as possible and to prevent death.^2^ Thus, patients at a low-risk of mortality may be candidates for home anticoagulant treatment or early hospital discharge. Patients at intermediate risk may require antithrombotic therapy and close clinical observation to escalate the treatment regime in case of clinical impairment, whereas patients at high-risk should proceed to prompt revascularization either by pharmacologic or percutaneous catheter-directed or surgical treatment.^2^

Thrombolysis combined with standard anticoagulation is potentially lifesaving. These procedures lead to faster improvements in pulmonary perfusion, hemodynamic defect, gas exchange, and right ventricular dysfunction. The foremost benefit is observed when thrombolysis is administrated within 48h of the onset of symptoms. Its efficacy decreases significantly after 7 days, but it may be beneficial up to 14 days after the onset of symptoms.^3^

The clinical advantage of thrombolytic therapy has been shown in patients with massive PE. High-risk patients who received thrombolytic therapy had a lower risk of all-causes and PE-related mortality than high-risk patients who did not receive it.^2,3^

However, in the meta-analysis performed by Quezada et al.^4^ showed that, despite hemodynamic instability, only 23% of high-risk patients underwent thrombolysis.

In thrombolytic therapy, the most significant dilemma is the risk of bleeding. In elderly patients, comorbidities and multidrug use further increase the risk of bleeding.^4^ This situation restricts the full indication of thrombolytics by clinicians, and as a result, there are few robust studies that have evaluated the efficacy and safety of thrombolysis in very elderly (>80 ys) patients with PE.

In this issue, Zengin et al.^5^ in a retrospective cohort study, have evaluated the effectiveness and safety of thrombolysis therapy in 148 aged octagenarian patients with PE. A conventional dose of the tissue plasminogen activator (tPA) was the standard lytic agent used for patients who sought medical care within 14 days of the onset of the symptoms.^2^ In that study, the authors showed a considerable in-hospital mortality rate of 19%. The thrombolytic group rate was significantly lower when compared to the non-thrombolytic group (10.5% *vs.* 24.2%, p=0.03). By contrast, the overall bleeding complications were significantly more frequent in the thrombolytic group, mainly determined by minor bleeding (35% *vs.* 13%, p<0.01). No difference was found when considering the major bleeding events (7% *vs* 5.5%, p=0.71).

Similarly, a retrospective cohort study,^6^ which included PE patients over 65 years of age, reported thrombolytic therapy associated with lower mortality and acceptable bleeding complication rates. Those findings may encourage clinicians to administer thrombolytic therapy with caution to elderly patients with PE.

However, one must bear in mind that PE is a life-threatening disease that becomes even more serious in the elderly population, with considerable in-hospital mortality. A portion of this mortality could be reduced by a careful thrombolysis indication. It is possible that the indication for thrombolysis in the elderly population could not be restricted only in hemodynamic instability, but this issue should be reviewed. The identification of elderly patients who are hemodynamically stable in the diagnosis, but who are at a high risk of early complications, is more challenging.

It is important to consider that in both types of studies, those for a risk stratification in PE patients using the Pulmonary Embolism Severity Index (PESI) and its Simplified form (sPESI), as well as studies with sPESI plus Troponin as a marker, are mainly based on data from a general population, where the much older patients were underrepresented.^7–10^ Therefore, the applicability of a risk stratification tool for elderly patients, developed based on data from younger populations, is questionable. Better risk stratification tools should be taken into consideration to improve the clinical management of the elderly (>65ys) and very elderly (>80) patients with suspected PE.

Another interesting finding to be highlighted in this study^11^ is a sample with only 30% of male patients included. Although there are no sex-specific comparative studies that have evaluated efficacy and safety in thrombolysis, it is important to stress the sex-related differences in platelet biology and coagulation reactions, resulting in different outcomes including bleeding complications.^11^ Thus, understanding sex-related differences with regard to antithrombotic therapy and anticoagulation should result in an individualized therapeutic approach for the prevention and treatment of different cardiovascular diseases.

Traditional contraindications to thrombolytic therapy should be considered relative in the scenario of high-risk PE, with the benefit-risk balance weighed in each individual case, especially in those over 80 years of age. The indication of an individualized thrombolysis, notably in the very elderly and frail population, should be reviewed to improve efficacy and safety, taking into account the sex.

To reduce the risk of bleeding in octagenarians, thrombolysis options with an individualized reduced dose of thrombolytic agents and lower doses by catheter-directed thrombolysis in that population need to be further studied.
